# Gastroenteritis Outbreaks Associated with the Emergence of the New GII.4 Sydney Norovirus Variant during the Epidemic of 2012/13 in Shenzhen City, China

**DOI:** 10.1371/journal.pone.0165880

**Published:** 2016-11-09

**Authors:** Yaqing He, Miao Jin, Kena Chen, Hailong Zhang, Hong Yang, Fei Zhuo, Dejian Zhao, Huatang Zeng, Xiangjie Yao, Zhen Zhang, Long Chen, Yuanping Zhou, Zhao-jun Duan

**Affiliations:** 1 Southern Medical University, Guangzhou, Guangdong, China; 2 Shenzhen Center for Disease Control and Prevention, Shenzhen, China; 3 Key Laboratory of Medical Virology and Viral Diseases; National Institute for Viral Disease Control and Prevention, China CDC, Beijing, China; 4 Shenzhen Luohu Center for Disease Control and Prevention, Shenzhen, China; University of Liverpool, UNITED KINGDOM

## Abstract

Noroviruses (NoVs) are the leading cause of gastroenteritis outbreaks in humans worldwide. Since late 2012, a new GII.4 variant Sydney 2012 has caused a significant increase in NoV epidemics in several countries. From November of 2012 to January of 2013, three gastroenteritis outbreaks occurred in two social welfare homes (Outbreaks A and B) and a factory (Outbreak C) in Shenzhen city of China. Feces and swabs were collected for laboratory tests for causative agents. While no bacterial pathogen was identified, all three outbreaks were caused by NoVs with detection rates of 26.2% (16/61) at Outbreak A, 35.2% (38/108) at Outbreak B), and 59.3% (16/27) at Outbreaks C. For Outbreak B, 25 of the 29 symptomatic individuals (86.2%) and 13 of the 79 asymptomatic individuals (16.5%) were found NoV-positive. For Outbreak C, an asymptomatic food handler was NoV-positive. All thirteen NoV sequences from the three outbreaks were classified into genogroup II and genotype 4 (GII.4), which we identified to be the GII.4 Sydney 2012 variant. The genome of two isolates from Outbreaks A and B were recombinant with the opening reading frame (ORF) 1 of GII.4 Osaka 2007 and ORF2 and 3 of the GII.4 New Orleans. Our study indicated that the GII.4 Sydney 2012 variant emerged and caused the outbreaks in China.

## Introduction

Noroviruses (NoVs), members of the *Norovirus* genus in the *Caliciviridae* family, are recognized as the most common viral cause of gastroenteritis outbreaks throughout the world [1–4], wherein they are responsible for > 90% of non-bacterial outbreaks of gastroenteritis worldwide [5]. In the United States, NoVs are estimated to cause 21-million illnesses each year, which results in 71,000 hospitalizations and 800 deaths [6,7]. In developing countries, NoVs are estimated to claim over 200, 000 deaths annually in children < 5 years of age [5]. Norovirus outbreaks occur frequently in semi-closed institutions, such as hospitals, nursing homes for the elderly, schools, prisons, restaurants, hotels, and cruise ships [8–13]. NoVs transmit via the consumption of contaminated food or water, through person-to-person contact, or by exposure to aerosols from vomitus [14–16]. NoVs are highly contagious owing to their ability to infect at low doses, their high stability in the environment, and by the limited short-term immunity hosts amount [17]. Accordingly, outbreaks of NoVs are extremely difficult to control.

NoVs are non-enveloped RNA viruses that contain a single-stranded, positive-sense, and polyadenylated RNA genome of ~ 7.5 kb in length [18]. The NoV genome consists of three open reading frames (ORFs 1–3). ORF1 encodes a polyprotein that is processed post-translation into six non-structural proteins, including an RNA-dependent RNA polymerase (RdRp) [19]. ORF2 and ORF3 encode the major (capsid protein, VP1) and minor (VP2) structural proteins, respectively [20]. The genomes of NoVs are highly diverse. Each belongs to one of six genogroups (GI–GVI) and each genogroup can be further stratified into one of more than thirty-six genotypes [21–23]. While GI, GII, and GIV NoVs can infect humans, the GII.4 genotype is attributed to causing both outbreaks and many sporadic cases [24].

Since the mid-1990s, global epidemics of NoV gastroenteritis have only been associated with the GII.4 genotype [1,25,13]. New variants tend to emerge from and supersede the then-predominant GII.4 variant every 2–3 years [24]. Several GII.4 variants have been attributed to numerous NoV gastroenteritis pandemics from 1996 to present, including the outbreaks of 95/96 US (1995–96) [25,4], Farmington Hills (2002–03) [13], Hunter (2004–05)[1], 2006b (2006–07) [26,27], New Orleans (2009–10) [28,29], and the most recent variant of Sydney 2012 (2012–13) [30–35]. Other GII.4 variants have caused regional epidemics—namely Henry 2001, Japan 2001, Asia 2003, 2006a, and Abeldoorn 2008 [36,24,37–42].

The recently emerged GII.4 variant, Sydney 2012, was firstly identified in Australia early in 2012, but also lead the increase of acute gastroenteritis outbreaks in the United States, France, Japan, United Kingdom, the Netherlands, New Zealand, and Hong Kong [30–35,43]. Furthermore, the Sydney 2012 variant was also responsible for three gastroenteritis outbreaks in two social welfare homes and a factory in Shenzhen, China, in 2012/13. Herein, we undertake epidemiological and laboratory investigations into these three gastroenteritis outbreaks caused by Sydney 2012 variant.

## Materials and Methods

### Ethics statement

The present study was approved by the Institutional Review Board of Shenzhen CDC. Written consent was obtained from both the symptomatic and asymptomatic individuals who participated in this study before collecting their stool samples and medical data.

### Epidemiological investigation

Cases were defined with ≥ 3 loose stools and /or vomiting in a 24-hour period during the outbreaks. A standardized questionnaire was developed for data collection of demographic data (sex and age), illness onset (symptoms, duration of symptoms) and potential risk factors (water and food consumption, patient contacts). We then collected stool and vomitus from the study set and took environmental swabs of vegetables and the chopping boards, bench surfaces, and cooking utensils used in the preparation of raw and cooked foods for laboratory diagnosis.

### RNA extraction and real-time PCR

A 10% stool suspension was prepared by mixing 0.1 g stool with 1.0 mL phosphate-buffered saline (pH 7.2). Viral RNA was extracted from the stool suspensions using the QiaAmp Viral RNA Mini Kit (Qiagen, Hilden, Germany) in accordance with the manufacturer’s protocol. Viral RNA was examined for GI and GII NoVs in a duplex format using the QuantiTect Probe RT-PCR kit (Qiagen, Hilden, Germany) on a 7500 Real-time PCR platform (Applied Biosystem). The final, 25-μL reaction mix consisted of 0.4 μM of each of the four primers (Cog1F, Cog1R, Cog2F, Cog2R)_and 0.2μM of each TaqMan Probe (Ring 1a, Ring1b, Ring 2) [44]. The cycling conditions were configured for a reverse transcription step at 50°C for 30 min, followed by a denaturation step at 95°C for 15 min and 40 cycles of amplification (denaturation at 95°C for 15 s and a combination of annealing and extension at 60°C for 1 min).

### Conventional reverse transcription (RT)-PCR

The capsid region used for NoV genotyping was amplified by conventional RT-PCR according to the protocol and primers (CoG2F, G2-SKR, G1-SKF, G1-SKR) reported by Yan *et al*. [45]. Thirteen stool samples determined to be NoV-positive by real-time PCR were submitted for RT-PCR.

### Amplification of full-length NoV genomes

The complete genomes of two of the earliest NoVs—JB031230049 and JB031230054—were amplified. Initially, cDNA was reverse transcribed from viral RNA (9.5 μL) using a modified oligo(dT)_20_ primer (V_3_NT_20_ in Table 1) and a SuperScript III first-strand synthesis system (Invitrogen, Carlsbad, CA). The total, 50-μL reaction mixture contained 5 μL 5× Ex Taq Buffer, 1 μL of each primer (20 pmol), 1 μL dNTP mix (10 mM), 0.5 μL Ex Taq enzyme (Takara, Japan), 5 μL cDNA, and 36.5 μL RNase-free water. CoG2F [45]and V_3_NT_20_ [46] primers were used to amplify the fragment from the ORF2 to the 3’ Poly A. The cycling conditions were configured as follows: initial denaturation at 94°C for 3 min, followed by 40 amplification cycles (94°C for 15 s, 62°C for 3 min, and 72°C 30s) and a final extension step at 72°C for 15 min. The ORF1 was divided into five overlapping fragments, which were amplified using the P290 and G2-SKR primers and four pairs of newly designed primer sets (Table 1). The conditions were as follows: initial denaturation at 94°C for 3 min, then 35 cycles (94°C for 45 s, 55°C for 45 s, and 72°C 1 min), and a final extension at 72°C 10 min. The fragment covering the ORF1/ORF2 overlap and amplified by the P290 [47] and G2-SKR [45] primers was also used to examine potential recombination.

**Table 1:** Primer sequences used for the present genome amplification.

Primer name	Sequence (5′→3′)	Polarity	Region^a^
Sydney2012-1F	GTGAATGAAGATGGCGTCTAAC	+	1–22
Sydney2012-1R	GGTAAATCCTAGCACCAAACCT	-	1045–1066
Sydney2012-2F	TGATTGGACCTTCGCAGGCATAG	+	871–893
Sydney2012-2R	TCTAGCCTCTCATGGAGTAACC	-	2055–2076
Sydney2012-3F	CATCCATGATGCCCTCAGGT	+	1660–1679
Sydney2012-3R	GATTTGCTTGATAGGGACTCCG	-	3148–3169
Sydney2012-4F	GCAACCGAAGAGGACTTCTGTGAAG	+	2813–2837
Sydney2012-4R	TGAGGAGCCAGTGGGCGATGGAAT	-	4497–4520
290 H	GATTACTCCAGGTGGGACTCCAC	+	4295–4317
290 I	GATTACTCCAGGTGGGACTCAAC		
290 J	GATTACTCCAGGTGGGATTCAAC		
290 K	GATTACTCCAGGTGGGATTCCAC		
G2SKR	CCACCTGCATAACCATTGTACAT	-	5367–5389
COG2F	CARGARBCNATGTTYAGRTGGATGAG	+	5003–5029
VN_3_T_20_	GAGTGACCGCGGCCGCT20	-	Poly A

^a^ Each sequence number of primer sets region is listed for Sydney 2012 strain (GenBank accession No. JX459908).

### DNA Sequencing and phylogenetic analysis

All PCR products were excised from the gel and purified using a QIAquick gel extraction kit (Qiagen, Hilden, Germany). All purified products were sequenced with the primers listed in Table 1 using the Big-Dye terminator cycle sequencing kit and the ABI Prism 310 Genetic Analyzer (Applied Biosystems Inc., Foster City, CA). The resulting NoV sequences were analyzed using CLUSTAL X (Version 1.83) followed by phylogenetic analysis using MEGA version 4.1. The statistical significance of the inferred phylogenies was estimated using a bootstrap analysis of 1,000 pseudoreplicate data sets. SimPlot software (Version 1.3) was used to align and compare sequences to identify potential recombination(s) among known genotypes of NoVs. The nucleotide sequences generated in this study were deposited in the GenBank (KJ995534–KJ995550 for short sequences within the capsid region and KJ955492–KJ955493 for complete genome sequences).

## Results

### Epidemiological investigation

**Fig 1:**
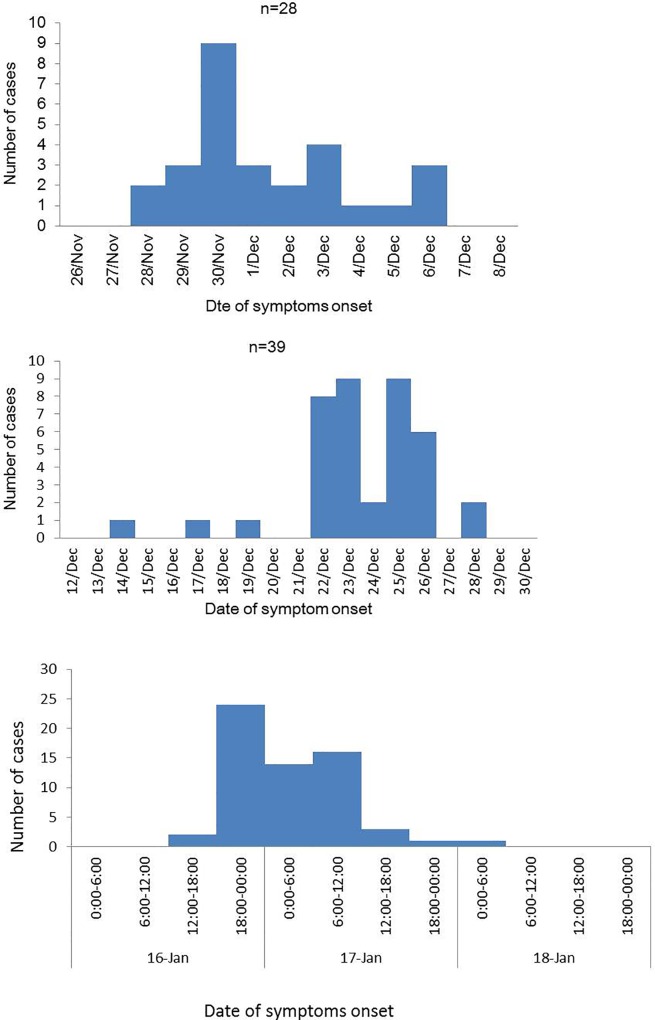
Date of illness onset associated with NoV Outbreaks A–C during the 2012/13 winter season in Shenzhen, China.

Among the three outbreaks of gastroenteritis in Shenzhen, China, during 2012/13 winter season, two arose in social welfare homes in the Luohu (Outbreak A) and Nanshan (Outbreak B) districts, while the epicenter of Outbreak C was in a factory in the Baoan district. Twenty-eight individuals were sick with acute gastroenteritis in the Outbreak A, including 22 residents and 6 healthcare workers. The index case occurred on November 28, 2012, with the outbreak peaking on November 30 (Fig 1). The most frequent symptoms were diarrhea (71.43%), followed by vomiting (60.71%), nausea (35.71%), abdominal pain (7.14%) and fever (3.57%; Table 2). No hospitalizations resulted from Outbreak A. Of the 28 infected persons, 19 were living on the fourth floor, 6 on the fifth, 2 on the third, and 1 on the sixth. The index case was an 82-year-old female living on the fourth floor.

**Table 2:** Frequency of reported clinical symptoms of gastroenteritis associated with Outbreaks A–C in Shenzhen between November and December 2013.

Symptom		Numbers of cases (%)	
	Outbreak A	Outbreak B	Outbreak C
**Diarrhea**	20 (71.4)	37 (94.9)	47 (77.0)
**Vomiting**	17 (60.7)	32 (82.1)	36 (59.0)
**Nausea**	10 (35.7)	2 (5.1%)	46 (75.4)
**Abdominal pain**	2 (7.1)	17 (43.6)	51 (83.6
**Fever**	1 (3.6)	1 (2.6)	12 (19.7)

In Outbreak B, 39 individuals were sick with gastroenteritis, including 33 residents, 5 healthcare workers, and 1 staff. The index case occurred on December 14, 2012. Outbreak B peaked between 23 and 25 of December (Fig 1). The most frequent symptoms were diarrhea (94.87%), followed by vomiting (82.05%), nausea (5.13%), abdominal pain (43.59%) and fever (2.56%; Table 2). We analyzed the attack rates among males and females stratified into age groups. Among the 33 sick residents, the attack rates were 18.33% (11/60) for males and 16.30% (22/135) for females, though this difference was not statistical significant (*X*^*2*^ = 0.12, *P* = 0.73). The ages of the 33 patients spanned from 48 to 92 years of age. The infection rate was highest in those > 80 years of age (54.55%, 18/33), followed by those aged 70–79 (27.27%, 9/33). The index case was an 83-year-old female living on the fifth floor of the healthcare center. She had 6–8 episodes of diarrhea on December 14 during her leave starting on December 8. She returned to the center on December 15 and had no symptoms of diarrhea on December 16. Thereafter, she visited entertainment rooms on the other floors, which culminated with at the highest attack rate on the fifth floor (31.25%; Table 3).

**Table 3:** Floor distribution of gastroenteritis cases of Outbreak B.

Floor	Total number	Numbers of cases	Affected
**2th**	42	9	21.43%
3th	37	9	24.32%
**4th**	27	2	7.41%
5th	32	10	31.25%
**6th**	38	1	2.63%
7th	19	2	10.63%

Seventy-six individuals were sick with acute gastroenteritis in Outbreak C. The index case occurred on January 16, 2013. Outbreak C peaked between January 16 and 17 (Fig 1). The common symptoms reported were pain (83.6%), nausea (5.13%), diarrhea (77.0%), vomiting (59.0%), and fever (19.7%; Table 2). Among the 414 staff, the attack rates were 15.63% (10/64) in males and 18.86% (66/350) in females—a different that was not statistically significant. The factory was a six-story building. The highest attack rate was 71.43% (5/7) on the sixth floor, followed by 25.32% (46/158) on the third, 16.13% (10/62) on the fourth, 11.83% (11/93) on the second, 4.94% (4/81) on the first floor. There were no cases on the fifth floor. Of the 76 infected, 43 (43/223, attack rate 19.28%) lived in the factory’s dormitory, 2 (2/15, attack rate 13.33%) lived in a dormitory external to the factory, and 31 (31/176, attack rate 17.61%) lived in private accommodation. There was no statistical significance among the attack rates when stratified by the location of dwelling or floors of the factory’s dormitory (Table 4).

**Table 4:** Floor distribution of gastroenteritis cases of Outbreak C.

Floor	Total number	Numbers of cases	Attack rate
**1th**	40	6	15.00%
2th	48	8	16.67%
**3th**	51	10	19.60%
4th	52	12	23.08%
**5th**	32	7	21.88%

### Virological investigation

All samples from the three outbreaks were negative for *E*. *coli*, *Salmonella*, *Shigella*, *Campylobacter*, *Y*. *enterocolittica*, rotavirus, adenovirus, and astrovirus. For Outbreak A, 14 of the 59 swabs and stool samples collected were NoV-positive, while all vomitus samples and environmental swabs were negative for NoV. For Outbreak B, 25 of the 29 symptomatic individuals (25/29, 86.2%) and 13 of the 79 asymptomatic individuals (13/79, 16.5%) were NoV-positive. For Outbreak C, 16 of the 27 stool swabs from symptomatic individuals (16/27, 59.26%) were NoV-positive; 18 environmental swabs and 2 food samples were negative for NoV; while one of the 7 asymptomatic food handlers was NoV-positive.

### Phylogenetic analysis

**Fig 2:**
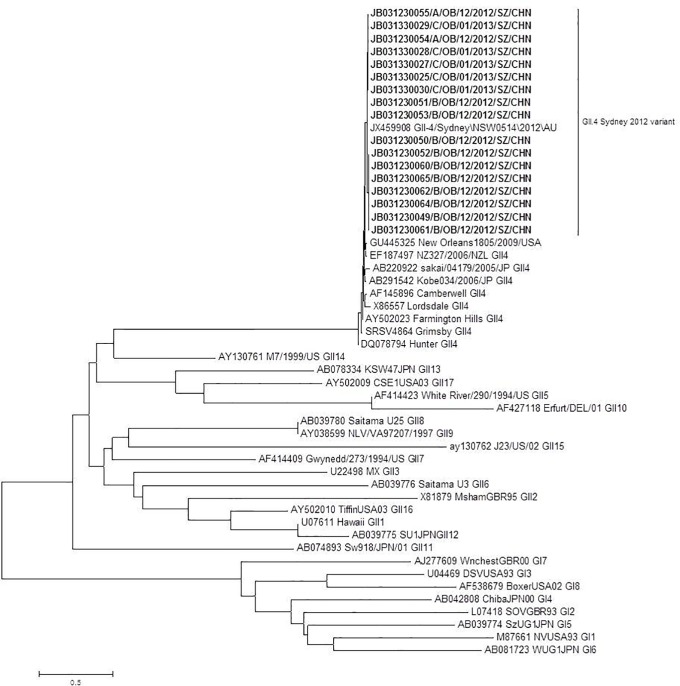
Phylogenetic tree based on the nucleotide sequences encoding the partial capsid (281 bp) associated with NoV outbreaks. Strains from the three studied outbreaks are denoted in bold. Phylogenetic analysis was performed using the neighbor-joining method (distance calculated by Kimura-2-parameter correction and pairwise deletion). Results were validated with 1,000 bootstrap pseudoreplicates.

**Fig 3:**
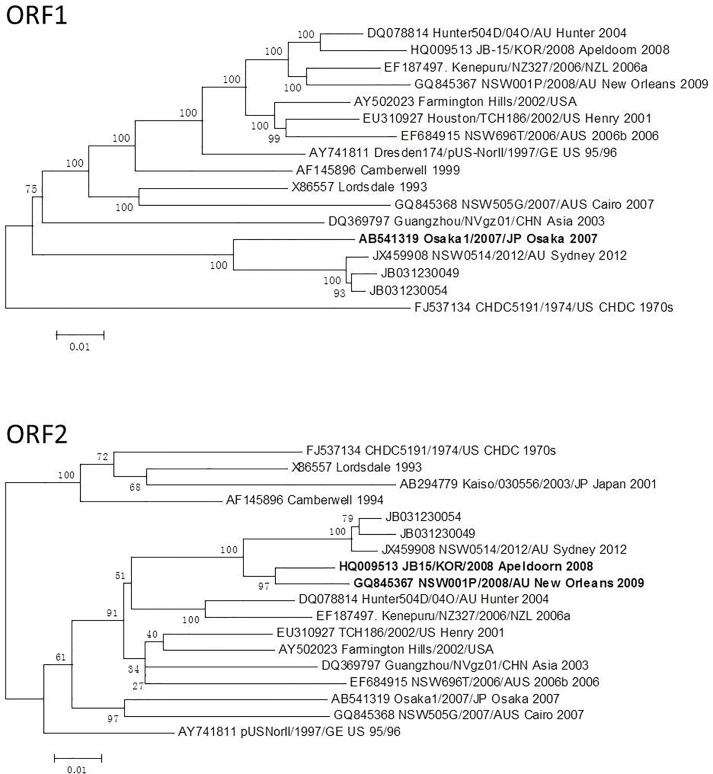
Changes in the phylogenetic locations of ORF1 and ORF2. JB031230054 strain from Outbreak A; JB031230049 strain from Outbreak B.

**Fig 4:**
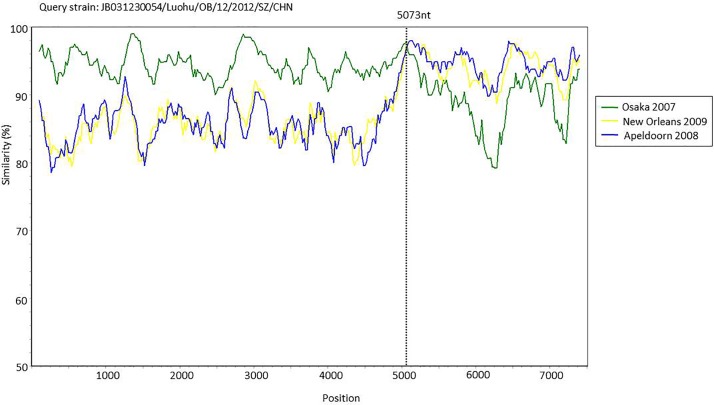
Simplot analysis for of putative, recombinant JB031230054 strain. Comparisons of genetic similarity between the strain JB031230054 and possible parental strains was made using SimPlot. The Y-axis represents the percentage of the nucleotide sequence similarity between the strain JB031230054 and other strains used for comparison. The X axis shows the relative nucleotide position along the full-length genome. The breakpoint positions are marked with dashed lines. A window size of 200 nucleotides with an increment of 20 was used.

Thirteen sequences from the three outbreaks were obtained and analyzed, including two from Outbreak A, six from Outbreak B, and five from Outbreak C. Phylogenetic analysis using a collection of reference sequences representing a variety of NoV genotypes showed that all thirteen of the sequences were classified into the genotype GII.4 and closely resembled the GII.4 Sydney 2012 variant (Genbank accession No. JX459908; Fig 2). To examine the potential recombination of the detected NoVs, the complete genome sequences of two isolates, JB031230054 (Outbreak A) and JB031230049 (Outbreak B) were amplified and sequenced. While both isolates clustered with the Sydney 2012 reference strain in three ORFs, their ORF1 clustered with the Osaka 2007 strain (GII.4 variant of 2007), while their ORFs 2&3 were clustered with the variants of Apeldoorn 2008 (GII.4 variant of 2008) and New Orleans (GII.4 variant of 2009), thereby suggesting a recombination event occurred during the overlap of ORF1/ORF2 from both isolates (Fig 3). To eliminate the possibility that the polymerase and capsid encoding genes were from two separate NoVs that co-infected the same patients, a fragment of ~1100 nucleotides in length, spanning both ORF1 and ORF2, was amplified from a stool sample. Simplot analyses of the junction regions between ORF1 and ORF2 confirmed the presence of recombinant strains and indicated a potential cross-over site where the recombination between ORF1 and ORF2 is likely to have occurred (Fig 4).

## Discussion

The present study describes three outbreaks of NoV that occurred in Shenzhen, China, during the epidemic season of 2012/13 caused by the new GII.4 variant Sydney 2012. During the past decade, new GII.4 variants have emerged and replaced previously predominant GII.4 strain every 2–3 years. Several global epidemics caused by different GII.4 variants in the winter season have been reported, including those that arose during the winter season of 1995/96 (caused by the 95/96 US variant), 2002/03 (caused by the Farmington Hills variant), 2004/06 (caused by the Hunter variant), 2006/07 (caused by the 2006b variant), and 2008/09 (caused by the New Orleans variant) [1,26,4,28,13]. The Sydney 2012 variant was first identified in Australia in March 2012, which had become global by late 2012 [30–35]. In the United States, the GII.4 Sydney variant caused a total of 141 (53%) of the 266 NoV outbreaks recorded from September to December of 2012 [48]. In Denmark, GII.4 Sydney accounted for 46 of the 106 (43%) NoV-positive samples typed from both surveillance and outbreaks from October to December of 2012 [49]. An increase in epidemics associated with the emergence of GII.4 Sydney was also reported in the UK, France, Japan, Australia, New Zealand by late 2012 [30–35,43].

Herein, we reported on three outbreaks caused by the same GII.4 Sydney NoV during the season of 2012/13 in Shenzhen, China. According to the outbreak data from several provincial CDCs in China, the GII.4 Sydney variant was responsible for 12 of the 13 other NoV outbreaks in the Guangdong and Jiangsu provinces in China between October of 2012 and March of 2013 (data not shown). These data suggested that the GII.4 Sydney strain has already caused an increase in NoV activities in China. The first case of GII.4 Sydney in China was reported in August under the national sporadic surveillance of NoV (data not shown).

NoV-associated gastroenteritis outbreaks are common in semi-closed settings, including hospitals, cruise ships, military camps, schools, and elderly care homes [8–13]. Over a quarter (27%) of NoV outbreaks, for example, are epicentered about healthcare settings [50], while long-term care facilities (elderly care homes, *etc*.) account for 50% of these NoV outbreaks occurring in a healthcare setting [50]. In our study, two of the three NoV outbreaks occurred in two social welfare centers that were funded by the local government as long-term care facilities for the elderly, while the remaining outbreak occurred in a factory that was a semi-close setting where many employees dwelled in communal dormitories.

A NoV outbreak is declared in accordance with Kaplan’s criteria, wherein (1) > 50% of affected persons must be afflicted with vomiting, (2) the mean or median incubation period is 24–48 hr, (3) that the mean or median illness duration is 12–60 hr, and (4) that no bacterial pathogens are isolated from stool cultures [51]. The epidemiological investigation in this study revealed that the vomiting rates of all three outbreaks were greater than 50% (60.7% for Outbreak A, 82.1% for Outbreak B, and 59.0% for Outbreak C). Asymptomatic infections are estimated to occur in about one-third of all studies investigating outbreaks involving volunteers. We detected 16% (13 out of 79) of asymptomatic persons who were NoV-positive in Outbreak C, including a food handler who could have been the source of this outbreak. Ozawa *et al*. reported that the prevalence of NoV detection in food handlers was 19% in different food-catering settings in Japan and that 73% of symptomatic and 7% of asymptomatic food handlers were positive for the NoV [52].

The evolution of GII.4 NoV variants is potentially driven by several factors [53]. In a similar manner to the influenza A virus, a new GII.4 variant tends to emerge every 2–3 year through antigenic drift. Furthermore, recombination is likely to be an important factor for the emergence of the GII.4 variants in a resemblance to the reassortment of genetic material by the influenza virus [54]. Intra-genotype recombination has frequently been reported in some GII.4 variants [41,55]. In this study, two genomes of the GII.4 Sydney 2012 variant from two outbreaks were analyzed for recombination. We found that ORF1 was derived from an Osaka 2007 virus, while the ORFs 2&3 were from the viruses responsible for Apeldoorn 2008 and New Orleans 2009 (Fig 3).

In conclusion, the emerging GII.4 Sydney 2012 variant caused three gastroenteritis outbreaks in the winter season of 2012/13 in Shenzhen in China. These outbreaks occurred during the same time frame when the GII.4 Sydney 2012 variant caused global epidemics. Two outbreaks occurred in social welfare centers for the elderly and were most likely due to an asymptomatic food handler. Our study highlights first the susceptibility and vulnerability of the elderly in the closed environment of a nursing home setting and secondly the need to improve sanitation practices by food handlers.
